# The Role of Health Literacy in COVID-19 Preventive Behaviors and Infection Risk Perception: Evidence from a Population-Based Sample of Essential Frontline Workers during the Lockdown in the Province of Prato (Tuscany, Italy)

**DOI:** 10.3390/ijerph182413386

**Published:** 2021-12-19

**Authors:** Vieri Lastrucci, Chiara Lorini, Marco Del Riccio, Eleonora Gori, Fabrizio Chiesi, Andrea Moscadelli, Beatrice Zanella, Sara Boccalini, Angela Bechini, Francesco Puggelli, Renzo Berti, Paolo Bonanni, Guglielmo Bonaccorsi

**Affiliations:** 1 Epidemiology Unit, Meyer Children’s University Hospital, Viale Gaetano Pieraccini 24, 50139 Florence, Italy; 2 Department of Health Sciences, University of Florence, Viale GB Morgagni 48, 50134 Florence, Italy; chiara.lorini@unifi.it (C.L.); beatrice.zanella@unifi.it (B.Z.); sara.boccalini@unifi.it (S.B.); angela.bechini@unifi.it (A.B.); paolo.bonanni@unifi.it (P.B.); guglielmo.bonaccorsi@unifi.it (G.B.); 3 Medical Specialization School of Hygiene and Preventive Medicine, University of Florence, Viale GB Morgagni 48, 50134 Florence, Italy; marco.delriccio@unifi.it (M.D.R.); eleonora.gori@unifi.it (E.G.); fabrizio.chiesi@unifi.it (F.C.); andrea.moscadelli@unifi.it (A.M.); 4 Management Department, Meyer Children’s University Hospital, Viale Gaetano Pieraccini 24, 50139 Florence, Italy; francesco.puggelli@meyer.it; 5 Department of Prevention, Azienda Sanitaria Locale Toscana Centro, Via Lavarone 3/5, 59100 Prato, Italy; renzo.berti@uslcentro.toscana.it

**Keywords:** health literacy, COVID-19, infection control, preventive measures, infection prevention behaviors, risk perception, population-based, essential frontline workers, knowledge, attitudes and practices

## Abstract

Background: The effectiveness of pandemic control measures requires a broad understanding from the population. This study aimed to evaluate the role played by health literacy (HL) in influencing the adherence to COVID-19 preventive measures and risk perception of essential frontline workers during the lockdown period. Methods: A cross-sectional survey was conducted on a population-based sample of frontline workers from Prato Province (Italy). Data on knowledge, attitudes and practices towards COVID-19 preventive measures and risk perception were collected. HL was measured with the HLS-EU-Q6 tool. Multivariate linear regression analyses were performed. Results: A total of 751 people participated in this study, and 56% of the sample showed a sufficient level of HL. In the multivariate models, HL resulted in being positively correlated with both knowledge (beta 0.32 for sufficient HL, 0.11 for problematic HL) and attitudes (beta 0.33 for sufficient HL, 0.17 for problematic HL) towards the importance of COVID-19 preventive measures. The HL level was not associated with the adoption of preventive behaviors and COVID-19 risk perception. Conclusions: HL may play a key role in maintaining a high adherence to infection prevention behaviors and may be a factor to take into account in the implementation of public health interventions in pandemic times.

## 1. Introduction

COVID-19 disease is caused by a highly transmissible and pathogenic coronavirus—SARS-CoV-2—that emerged in 2019 and rapidly became a major public health issue. SARS-CoV-2 is mainly transmitted from person to person via respiratory droplets [1]. Since early 2020, countries around the world have faced extraordinary challenges to effectively slow the spread of COVID-19 and sustain their healthcare systems. Until early 2021, there were no vaccines for COVID-19, and countries were reliant on the implementation of large-scale collective measures to prevent disease transmission such as the prohibition of large-scale events, stay-at-home orders and national/local lockdowns. Besides collective measures, people were asked to follow individual preventive measures that included handwashing, wearing face masks, respecting physical distancing and other precautions such as keeping rooms well ventilated, avoiding gatherings and coughing into a bent elbow or tissue. Large sensitization campaigns were launched in order to explain these preventive measures to populations [2].

In the Tuscany region, the first case of COVID-19 was observed at the end of February 2020. The region and the province of Prato—the setting of the present study—had a slower epidemic trend than the Italian average [3]. To contain the circulation of SARS-CoV-2 during the first wave of the epidemic, the Italian government adopted a national lockdown from 11 March to 3 May 2020, which suspended all non-essential services and activities, including schools, and imposed a stay-at-home order prohibiting all non-essential travel and contact with others. Besides these measures, physical distancing rules and the obligation to wear a face mask when leaving home were introduced [4,5].

It is widely known that people differ in their ability to understand, access and act on health information and to make informed health decisions—a set of knowledge and competences that commonly falls under the term of health literacy (HL). Indeed, HL can be defined as “the ability of citizens to make sound decisions concerning health in daily life—at home, at work, in healthcare, at the market place and in the political arena” [6]. Recently, HL has emerged as one of the strongest determinants of health outcomes and behaviors and as an underlying factor of several health inequalities across different age, ethnicity and socioeconomic groups [7,8,9].

The adherence to various preventive and health-related behaviors has been reported to be positively associated with the level of HL [10,11,12,13,14]. As for COVID-19 preventive measures, to date, only few studies have evaluated the role of HL in influencing the adherence to such behaviors. A study by Li and Liu [15] found that the level at which the public engages in preventive measures during the pandemic is widely influenced by the level of HL. Similarly, Hong et al. [16] found that HL was significantly related to infection preventive behaviors in a sample of undergraduate students majoring in healthcare. At the individual and community levels, HL can play a relevant role in influencing the effectiveness of infection control measures promoted during the COVID-19 pandemic as these measures mostly rely on the ability of individuals to understand and adopt the correct preventive behaviors and to respect the rapidly evolving public health measures [17]. Furthermore, the role of HL appears to be even more relevant in essential frontline workers, especially those from the health and social care sectors, because they are potentially at higher risk of exposure to infection and because they may also have a role in correcting misconceptions and propagating correct healthcare information to the population [5,18,19,20].

Therefore, the aim of the present study was to evaluate the role played by HL in influencing the adherence to COVID-19 preventive measures and risk perception in a population-based sample of essential frontline workers during the lockdown period. In particular, this study was carried out on a representative sample of workers of the Civil Protection and public employees of the municipalities of the province of Prato (Tuscany region, Italy); the relationship between the level of health literacy and the knowledge, attitudes and practices towards COVID-19 prevention measures was evaluated. Furthermore, this study explored the association between the level of HL and COVID-19 infection risk perception.

## 2. Materials and Methods

This study was approved by the local ethics committee and was conducted according to the principles described in the Declaration of Helsinki.

### 2.1. Study Population

A cross-sectional survey was carried out on a population-based sample in the province of Prato (Tuscany, Italy). The study population was composed of individuals aged 18 years or older involved in different activities considered essential during the lockdown period of the first COVID-19 pandemic wave in Italy (11 March to 3 May 2020). In particular, all the people belonging to the following population groups in the province of Prato were invited to participate: workers and volunteers of the Civil Protection, public employees of the province of Prato and public employees of the municipalities constituting Prato Province (i.e., Cantagallo, Carmignano, Montemurlo, Poggio a Caiano, Vaiano and Vernio). All the workers working from home were excluded from the study.

### 2.2. Data Collection and Measurements

The study was conducted as part of a COVID-19 serological testing campaign. Eligible persons (*n* = 778) were invited to join the study by the head of each service, who provided them with an informative letter. On the testing day, participants who gave their consent were asked to fill a paper-based self-administered questionnaire. Questionnaires were distributed and collected by the research team; during the testing day, researchers were available for providing additional information and explanations concerning the research and the survey. Data were collected between May and June of 2020. The questionnaire consisted of 67 questions (average completion time of 25 min) covering the following topics: sociodemographic characteristics; health literacy; health conditions increasing the risk of severe illness from COVID-19; knowledge, attitudes and practices (KAP) towards COVID-19 prevention measures; COVID-19 risk perception.

As for sociodemographic characteristics, the following variables were collected: age, sex, education level, nationality.

The Italian version of the 6-item European Health Literacy Survey Questionnaire (HLS-EU-Q6) was used to assess the HL level [21,22,23]. HLS-EU-Q6 is a self-reported tool with the following Likert-type response options: very easy (score = 4), fairly easy (score = 3), fairly difficult (score = 2), very difficult (score = 1) and do not know or refusal (coded as missing). The scale’s final score was the mean value of the scores of all the items and varied between 1 and 4. Only respondents who answered at least 5 items were considered. According to the final score (x), three possible levels of HL were defined: inadequate HL (1 ≤ x ≤ 2); problematic HL (2 < x < 3); sufficient HL (3 ≤ x ≤ 4).

As for health conditions that may increase the risk of severe illness from COVID-19, the presence of the following underlying conditions was investigated (response options: yes, no): diabetes, overweight and obesity, heart diseases, pulmonary diseases, diseases of the immune system, chronic kidney diseases, chronic liver diseases, organ or bone marrow transplantation, chronic neurological diseases, oncological diseases in the last 5 years, hematological diseases, pregnancy status, major surgery intervention that required general anesthesia during the last year, current smoker. Furthermore, participants were asked if people aged 64 years or older or people with chronic diseases lived in their household.

KAP towards COVID-19 preventive measures and COVID-19 risk perception were investigated using items developed by the research group to fulfill the research questions. In particular, the item generation process was as follows: review of the literature, and Delphi procedure within the research group to obtain the final shared version of the items. Pretesting was not conducted due to the restrictive measures adopted in Italy in that period and due to the need to rapidly conduct the survey. KAP were investigated with Likert scale questions with five response options. As for the knowledge on the COVID-19 prevention measures, participants were asked to self-report their general knowledge of the measures required for protecting themselves from/preventing the transmission of COVID-19 (response options: very poor, poor, fair, good, very good; score 1 to 5). As for the attitudes towards COVID-19 preventive measures, participants were asked to rate the importance of the following preventive measures (response options: low importance, slightly important, neutral, moderately important, very important; score 1 to 5): i. washing hands on all recommended occasions; ii. wearing a face mask; iii. staying at home as much as possible; iv. keeping a physical distance of at least one meter from other people; v. avoiding meeting friends and family members from another household, except for reasons of extreme necessity. Lastly, as for COVID-19 prevention practices, participants were asked to self-report their adherence to the basic recommendations for COVID-19 prevention (response options: never, almost never, occasionally/sometimes, almost every time, every time; score 1 to 5).

COVID-19 risk perception was assessed asking participants the following three questions (response options: not at all, slightly, moderately, very, extremely; score 1 to 5): i. How likely do you think you will contract COVID-19? ii. How dangerous do you think COVID-19 is for your health? iii. How afraid are you that your family members could contract COVID-19?

### 2.3. Statistical Analysis

Data were presented as percentage, mean (±standard deviation—SD) or median (interquartile range—IQR), as appropriate. The normality of numerical variables was tested using the Kolmogorov–Smirnov test. For the items related to the attitudes towards COVID-19 prevention measures, the mean was calculated for each subject as an overall summary trend index (“overall attitude score”). The same approach was used for the items related to COVID-19 risk perception (“overall risk perception score”). For all the scores of the items related to KAP towards COVID-19 preventive measures and COVID-19 risk perception, both ceiling and floor effects were checked, i.e., whether 15% of the participants scored the highest and the lowest score, respectively.

The association between the subgroups and the collected variables was tested using Fisher’s exact test, the Mann–Whitney U test or the Kruskal–Wallis test. Spearman correlation analysis was used to test correlations between the HLS-EU-Q6 score and the responses to Likert-type items.

Multivariate linear regression analyses were performed to assess the role of the collected variables in predicting the KAP towards COVID-19 preventive measures and COVID-19 risk perception. In particular, four different models were executed, considering the score on the knowledge item, the overall attitude score, the score on the behaviors item and the overall risk perception score as dependent variables (outcomes). For each model, the independent variables (predictors) were those that were statistically significantly associated with the dependent variables in the univariate analyses. Using a backward stepwise procedure, the final models of each of the four multivariate regression analyses were obtained.

All the analyses were run using IBM SPSS 27 (IBM Corp., Armonk, NY, USA), considering an alpha level of 0.05 as significant.

## 3. Results

Overall, 751 people filled out the questionnaire, with a study participation rate of 96.5% (95.8% and 96.9% in the public employee and Civil Protection groups, respectively). Participants working/volunteering in the Civil Protection totaled 502 (66.8% of the total sample), and participants working as public employees in the municipalities of the province of Prato totaled 249 (31.2%). The median age of the total sample was 50 years (IQR: 36–60; range: 16–84), with a significant difference between the two subgroups of essential workers (*p* < 0.001): 53 years in the Civil Protection group (IQR: 33.5–64; range: 16–84) and 48 years for public employees of municipalities in the province (IQR: 48–56; range: 21–74).

Descriptive analyses of sociodemographics, risk conditions and HL levels are reported in Table 1. Participants were mostly males (59%), younger than 65 years old (83.5%), Italian (98.5%), with a high education level (high school or higher: 60.6%), with sufficient HL (56%) and never smokers (54.2%). Moreover, 27.4% of participants lived with people older than 64 years or with people with chronic diseases, and 25.3% presented at least one of the investigated health conditions that may increase the risk of severe illness from COVID-19 (Table 1).

Compared with public employees of municipalities in the province, participants from the Civil Protection were more frequently males, older than 64 years old, less educated and smokers and had a higher percentage of sufficient HL (Table 1). The median HL score in the sample was 3 (IQR: 2.6–3.3; range: 1–4) without statistically significant differences between the two groups.

The descriptive analysis of the items related to KAP towards COVID-19 preventive measures and COVID-19 risk perception is reported in Table 2. A high level of self-reported knowledge (mean: 4.31 ± 0.787; median: 4), attitudes (for each item, mean higher than 4.44 and median equal to 5; overall attitude score, mean: 4.65 ± 0.513, median: 5) and adopted practices (mean: 4.52 ± 0.639; median: 5) towards COVID-19 preventive measures was observed. In terms of COVID-19 risk perception, a high score was observed for the item related to the fear that a family member could contract the disease (mean: 4.52 ± 0.639; median: 5), while for the other risk perception items (“How likely do you think you will contract COVID-19?”; “How dangerous do you think COVID-19 is for your health?”), a lower risk perception level was observed (mean: 3.27 ± 1.123, median: 3, and mean: 3.95 ± 1.092, median: 4, respectively). A ceiling effect was observed for the item related to the knowledge of preventive measures (48.2% of participants had the highest score), for the overall attitude score (50% had the highest score) and for the item related to the adoption of practices for COVID-19 prevention (57.5% had the highest score).

The results of the correlation analysis between HL (HLS-EU-Q6 score) and KAP towards preventive measures and COVID-19 risk perception items are reported in Table 3. All the items related to KAP toward COVID-19 preventive measures (with the exception of the item “to wash your hands on all recommended occasions”) were significantly and positively correlated with the HLS-EU-Q6 score in the total sample (Table 3). As for the COVID-19 risk perception items, no significant associations were found with the HLS-EU-Q6 score (Table 3).

The variables significantly associated with the outcome variables (*p* < 0.05) were included in the four multivariate regression models (one for each outcome variable). The final models were obtained applying the backward stepwise procedure. In Table 4, the final models are described.

Knowledge of COVID-19 preventive measures was significantly and negatively predicted by having one or more health conditions that may increase the risk of severe illness from COVID-19 (beta: −0.18); moreover, sufficient HL (with respect to inadequate HL) and smoking more than 20 cigarettes/day (with respect to being never smokers) significantly and positively predicted a higher level of knowledge of COVID-19 preventive measures (beta: 0.32 for sufficient HL; 0.23 for smoking more than 20 cigarettes/day). The overall attitude score towards the importance of COVID-19 preventive measures was significantly and positively predicted by female sex (beta: 0.11) and HL level (compared with inadequate HL, beta: 0.17 and 0.33 for problematic and sufficient HL, respectively), while it was negatively predicted by being public employees of municipalities in the province (beta: −0.12, compared with workers of the Civil Protection). The adoption of practices for COVID-19 prevention was significantly and positively predicted by being a female (beta: 0.15) and smoking more than 20 cigarettes/day (beta: 0.19, compared with being never smokers), while it was negatively predicted by being a public employee of municipalities in the province (beta: −0.12, compared with workers of the Civil Protection). In terms of the overall COVID-19 risk perception score, it was significantly and positively predicted by having one or more health conditions that may increase the risk of severe illness from COVID-19 (beta: 0.15) and by foreign nationality (beta: 0.53).

## 4. Discussion

The aim of this study was to explore the role of health literacy in predicting KAP towards COVID-19 prevention measures and the infection risk perception in a population-based sample of individuals who performed essential activities during the first Italian general lockdown (March–May 2020). In particular, this study was conducted on a representative sample of workers of the Civil Protection and public employees of essential services of the municipalities of the province of Prato, Italy. The results of multivariate linear regression analyses showed that the level of HL of participants positively predicted the level of knowledge on COVID-19 prevention measures and the attitude towards the importance of adopting those measures, whereas the level of HL did not significantly predict the implementation of COVID-19 prevention behaviors. Lastly, the level of HL was not significantly associated with COVID-19 risk perception, while having one or more health conditions that may increase the risk of severe illness from COVID-19 and being of a foreign nationality were significantly associated with increased COVID-19 risk perception.

As for the characteristics of the sample, there were several significant differences between the two groups of essential workers. Such differences most likely reflect the different specificities of the work typology analyzed as the type of jobs constituting the two groups are related to different backgrounds and inclinations and may require different qualifications (e.g., it is a mandatory requirement to have at least a high school diploma for most of the jobs in the municipalities). Furthermore, although, during the first lockdown, people with chronic diseases were advised to stay at home, a relevant proportion of the sample reported a chronic disease. One possible explanation for this finding may be the lack of available backup staff to take over the responsibilities of workers with chronic diseases. Another possible concurrent explanation is that the chronic diseases reported were mild for some participants; however, this is not possible to ascertain as the severity of the chronic diseases was not investigated in our study.

Overall, in our population, the level of knowledge, attitudes and practices concerning COVID-19 preventive measures was considerably high. In this regard, it should be highlighted that the population examined in this study was composed of workers in essential services that were required to interact with other people (e.g., colleagues, users and patients) during the first Italian lockdown; therefore, in such a population group and public health emergency period, the correct knowledge and management of COVID-19 prevention measures were essential both for protecting themselves from contracting/transmitting COVID-19 and for providing correct health information to the general population. The nature of the population group examined may also likely explain the registered high proportion of people with an adequate level of HL, which is considerably higher than that reported in the general population [22,23,24,25,26]; indeed, the population group was composed of people who are employed and whose position mostly requires a higher education level and a background in the health and social care fields.

As for the relationship between health literacy and COVID-19 prevention measures, in our study, the level of HL resulted in being positively associated with the level of knowledge and attitudes toward COVID-19 preventive measures. In recent years, HL has increasingly gained attention in public health studies as one of the key determinants of health [9,27,28]. However, research on this topic mainly focused on the role of HL in influencing health outcomes or unhealthy lifestyles [27,28,29], and less attention has been paid to its role in influencing the adoption of preventive behaviors in the context of public health emergencies, such as a pandemic [15,16,17]. Nonetheless, public health measures—both individual and collective—that have to be implemented during an epidemic require a high degree of understanding and involvement of the population in order to be effective, as most of them require respecting restrictions and adopting burdensome behaviors for the public good. In this regard, HL may play a key role in the adherence to infection prevention behaviors and may be a factor to take into account in the implementation of public health interventions and campaigns in pandemic times.

Interestingly, the level of HL was not associated with the implementation of COVID-19 prevention measures in our study. A possible explanation for this finding is that, considering the context and the population group studied (i.e., the first lockdown period and workers in essential services), it may be possible that COVID-19 prevention measures were implemented by participants independent of their ability to access, understand, appraise and apply health information (i.e., their level of HL); in confronting, for the first time, such a unique public health emergency, it is plausible that other factors may have caused participants to strictly adopt prevention behaviors, such as fear of the contagion, work organization policies and public/social pressure. Indeed, the level of adoption of COVID-19 prevention measures was considerably high, with a high proportion of participants in the study reporting the maximum score on this item. This may have reduced the possibility of discriminating the adoption of prevention measures by level of HL (i.e., ceiling effect), and thus it may have led to absent correlations. The lack of impact of HL on COVID-19 prevention practices highlights that a different approach should be adopted to increase their adoption in the early stages of the pandemic; this suggests that empowering interventions and strategies based on behavior change communication may be more effective in improving the adherence to COVID-19 prevention practices. However, in subsequent phases of the pandemic, it should be pointed out that the role of the above reported factors in influencing the adoption of prevention behaviors may be reduced as the attention on prevention goes down—so-called “pandemic fatigue” [30]; in such later phases of the pandemic, the adoption of prevention behaviors could be more linked with the actual level of knowledge and attitudes towards COVID-19 prevention measures, and thus with the HL level. Further studies considering different phases of the pandemic are needed in order to evaluate the relationship between HL and the adoption of COVID-19 prevention measures.

Smoking status resulted in being associated with better knowledge of and higher adherence to COVID-19 prevention practices in our study. A possible explanation for this association may be related to the fact that, in the early stages of the pandemic, there were claims that smoking was associated with severe disease in COVID-19 patients. Although such claims remain to be confirmed to date [31], in the early phases of the pandemic, they may have influenced smokers to deepen their knowledge and to increase their adherence to prevention practices.

The SARS-CoV-2 infection risk perception of participants was not related to their level of HL in our study. Contrasting results are present in the literature concerning the relationship between HL and risk perception, with some studies reporting an inverse correlation [32,33], and others reporting no associations [34]. Risk perception is a complex concept; indeed, the assessment of risk is influenced by elements belonging not only to the rationality domains (e.g., specific health cognition) but also to other domains such as individual past experiences, personality and emotional factors and level of trust in society [35,36]. This variegated nature of the elements influencing risk perception may explain the contrasting results concerning its relationship with HL. It may be hypothesized that, depending on the topic in which health risk perception is investigated, the role of these elements may vary, thus influencing the potential role of HL. In the case of the first COVID-19 pandemic wave, it is plausible that elements concerning emotional factors—such as the fear and anxiety concerning a novel disease—personal history and level of societal trust (also influenced by the uncertainty of the situation, the contrasting and rapidly evolving public health messages and measures, and fake news that undermined the credibility of institutions) may have prevailed over knowledge and understanding of the disease in influencing risk perception. Interestingly, participants with a foreign nationality showed a higher risk perception; this may be linked to fear of the disease due to more vulnerable socioeconomic positions. However, in this regard, it should be pointed out that the regional public health system provides healthcare free of charge for the entire population [37].

The present study has several strengths and limitations. This is one of the first studies assessing the role of HL in infection prevention behaviors and risk perception during a pandemic. Furthermore, the study population can be considered representative of the entire study area for the selected population groups. As for the study limitations, first, the study was limited to a specific phase of the COVID-19 outbreak, and thus our findings are restricted to the very first phase of the pandemic and cannot be generalized to other phases of the pandemic. Second, the study consisted of a sample of specific groups of essential workers, which is not necessarily representative of the general population or of other types of essential workers; thus, the findings can be considered to have external validity only for the population subgroups considered. Third, the study did not consider the exact job tasks performed by the participants; thus, further research considering this aspect is warranted as job tasks may have a role in influencing outcomes—particularly prevention practices and risk perception. Fourth, knowledge, attitudes and practices towards COVID-19 prevention were self-reported by the participants; therefore, the results may have suffered from social desirability bias of the participants. However, it should be underlined that the survey was self-administered and completely anonymous. Lastly, a high proportion of participants in the study reported the maximum scores in the outcome variables. This “ceiling effect” made discrimination among participants among the top end of the scale impossible and may have led to attenuated or absent correlations between HL and outcome variables.

## 5. Conclusions

The effectiveness of pandemic control measures requires broad understanding and support from the population. The present study investigated the role of HL in influencing the knowledge, attitudes and practices towards COVID-19 prevention and risk perception in a representative sample of workers in essential public services during the first lockdown period. The findings of the study show that the HL skills of participants were positively correlated with the knowledge and attitudes towards infection prevention measures, while health literacy did not predict adherence to COVID-19 infection prevention measures: the actual adherence to infection prevention behaviors was high but not related to HL. These findings suggest that, in the first phase of the pandemic, prevention behaviors may be followed independent of the ability to understand and appraise health information. However, the positive association between HL and knowledge and attitudes towards COVID-19 prevention measures suggests that, in the later phases of the pandemic—when fears of the disease and attention towards prevention behaviors may decrease—HL may be a key determinant in maintaining a high level of adherence to prevention measures. Thus, interventions based on behavior change communication rather than health literacy may improve adherence to infection prevention measures in the early stages of a pandemic. In the later phases of a pandemic, programs and interventions aimed at increasing the level of HL in frontline essential workers may be needed for maintaining and enhancing both their safety in the workplace and skills in guiding the general population on the correct prevention measures; however, further studies carried out in different periods of the pandemic are needed to elucidate this aspect. Furthermore, as the level of comprehension on prevention measures seems to be related to HL skills, the findings of this study highlight the need to take into account people’s HL in the design of infection prevention campaigns.

## Figures and Tables

**Table 1:** Sociodemographics, health literacy level and risk conditions of the whole sample and in Civil Protection and public employee subgroups (*n* = 751 participants, year 2020).

Variables	Whole Sample *n* (%)	Civil Protection 502 (66.8%)	Public Employees 249 (31.2%)	*p*
Sex				<0.001
Females	308 (41%)	175 (34.9%)	133 (53.4%)	
Males	443 (59%)	327 (65.1%)	116 (46.6%)	
Age				<0.001
≤64 years	626 (83.5%)	380 (75.8%)	246 (98.8%)	
>64 years	124 (16.5%)	121 (24.2%)	3 (1.2%)	
Nationality				0.019
Italian	740 (98.5%)	491 (97.8%)	249 (100%)	
Other	11 (1.5%)	11 (2.2%)	0	
Education level				<0.001
Primary school or less	53 (7.1%)	52 (10.4%)	1 (0.4%)	
Lower secondary school	243 (32.4%)	209 (41.6%)	34 (13.7%)	
High school	310 (41.3%)	194 (38.6%)	116 (46.6%)	
Bachelor degree or higher	145 (19.3%)	47 (9.4%)	98 (39.4%)	
Health literacy (HLS-EU-Q6) *				0.046
Inadequate	50 (7.7%)	35 (8.1%)	15 (7%)	
Problematic	235 (36.3%)	143 (33%)	92 (43%)	
Sufficient	362 (56%)	255 (58.9%)	107 (50%)	
Living with people aged 64 years or older or with people with chronic diseases	206 (27.4%)	149 (29.7%)	57 (22.9%)	0.056
Risk conditions for severe COVID-19				
diabetes	31 (4.1%)	23 (4.6%)	8 (3.2%)	0.535
overweight/obesity	54 (7.2%)	35 (7%)	19 (7.6%)	0.714
heart diseases	28 (3.7%)	25 (5%)	3 (1.2%)	0.038
pulmonary diseases	29 (3.9%)	17 (3.4%)	12 (4.8%)	0.272
diseases of the immune system	27 (3.6%)	13 (2.6%)	14 (5.6%)	0.102
chronic kidney diseases	4 (0.5%)	4 (0.8%)	0	0.384
chronic liver diseases	2 (0.3%)	2 (0.4%)	0	0.803
organ or bone marrow transplant	2 (0.3%)	1 (0.2%)	1 (0.4%)	0.405
chronic neurological diseases	11 (1.5%)	9 (1.8%)	2 (0.8%)	0.192
oncological diseases (last 5 years)	14 (1.9%)	10 (2%)	4 (1.6%)	0.427
hematological diseases	5 (0.7%)	2 (0.4%)	3 (1.2%)	0.059
pregnancy	8 (1.1%)	3 (0.6%)	5 (2%)	0.089
major surgery with general anesthesia (last year)	29 (3.9%)	23 (4.6%)	6 (2.4%)	0.284
at least one health risk conditions	190 (25.3%)	134 (26.7%)	56 (22.5%)	0.123
Smoking habits				<0.001
Never smokers	407 (54.2%)	254 (50.6%)	153 (61.4%)	
Current smokers, less than 10 cigarettes/day	88 (11.7%)	65 (12.9%)	23 (9.2%)	
Current smokers, 10–20 cigarettes/day	13 (1.7%)	7 (1.4%)	6 (2.4%)	
Current smokers, more than 20 cigarettes/day	87 (11.6%)	74 (14.7%)	13 (5.2%)	
Former smokers	156 (20.8%)	102 (20.3%)	54 (21.7%)	

* Missing value: 104 in the whole sample, 69 among Civil Protection, 35 among public employees.

**Table 2:** Knowledge, attitudes and practices towards COVID-19 preventive measures and COVID-19 risk perception of the whole sample and in Civil Protection and public employee subgroups (*n* = 751 participants, year 2020).

Area	Items	Whole Sample		Civil Protection		Public Employees		*p*
		Mean ± SD	Median (IQ Range)	Mean ± SD	Median (IQ Range)	Mean ± SD	Median (IQ Range)	
Knowledge	Knowledge of prevention measures to protect from and avoid transmitting COVID-19, from 1 (very poor) to 5 (very good)	4.31 ± 0.787	4 (4–5)	4.35 ± 0.795	5 (4–5)	4.22 ± 0.766	4 (4–5)	0.008
Attitudes	Importance of the following measures to protect from and avoid transmitting COVID-19, from 1 (low importance) to 5 (very important):							
	washing hands on all recommended occasions	4.85 ± 0.446	5 (5–5)	4.85 ± 0.467	5 (5–5)	4.87 ± 0.401	5 (5–5)	0.428
	wearing a face mask	4.79 ± 0.557	5 (5–5)	4.82 ± 0.546	5 (5–5)	4.72 ± 0.575	5 (5–5)	0.004
	staying at home as much as possible	4.44 ± 0.923	5 (4–5)	4.51 ± 0.911	4 (4–5)	4.29 ± 0.931	5 (4–5)	<0.001
	keeping a distance of at least one meter from other people	4.76 ± 0.588	5 (5–5)	4.77 ± 0.613	5 (5–5)	4.75 ± 0.535	5 (5–5)	0.097
	avoiding meeting friends/family members from another household, except for reasons of necessity	4.40 ± 0.928	5 (4–5)	4.49 ± 0.897	5 (4–5)	4.22 ± 0.966	5 (4–5)	<0.001
	Overall attitude score	4.65 ± 0.513	5 (4.4–5)	4.69 ± 0.520	5 (4.6–5)	4.57 ± 0.489	4.8 (4.2–5)	<0.001
Practices *	Adherence to the basic recommendations for COVID-19 prevention, from 1 (never) to 5 (every time)	4.52 ± 0.639	5 (4–5)	4.57 ± 0.659	5 (4–5)	4.43 ± 0.587	4 (4–5)	<0.001
Risk perception	From 1 (not at all) to 5 (extremely), how likely do you think you will contract COVID-19?	3.27 ± 1.123	3 (3–4)	3.33 ±1.198	3 (3–4)	3.15 ± 0.947	3 (3–4)	0.016
	From 1 (not at all) to 5 (extremely), how dangerous do you think COVID-19 is for your health?	3.95 ± 1.092	4 (3–5)	3.99 ± 1.136	4 (3–5)	3.87 ± 0.996	4 (3–5)	0.031
	From 1 (not at all) to 5 (extremely), how afraid are you that your family members could contract COVID-19?	4.52 ± 0.639	5 (4–5)	3.60 ± 1.145	4 (3–4)	3.65 ± 1.029	4 (3–4)	0.908
	Overall risk perception score	3.61 ± 0.822	3.7 (3–4.3)	3.64 ± 0.868	3.67 (3–4.33)	3.55 ±0.721	3.67 (3–4)	0.088

* 7 missing values.

**Table 3:** Spearman correlation analysis between the items related to knowledge, attitudes and practices towards COVID-19 preventive measures and COVID-19 risk perception and the HLS-EU-Q6 score for the whole sample and in Civil Protection and public employee subgroups (*n* = 751 participants, year 2020).

Area	Items	Whole Sample Rho Values	Civil Protection Rho Values	Public Employees Rho Values
Knowledge	Knowledge of prevention measures to protect from and avoid transmitting COVID-19	0.206 *	0.159 *	0.288 *
Attitudes	Importance of the following measures to protect from and avoid transmitting COVID-19:			
	washing hands on all recommended occasions	0.074 °	0.102 ^#^	0.029 °
	wearing a face mask	0.176 *	0.195 *	0.139 ^#^
	staying at home as much as possible	0.173 *	0.171 *	0.168 ^#^
	keeping a distance of at least one meter from other people	0.162 *	0.162 *	0.158 ^#^
	avoiding meeting friends/family members from another household, except for reasons of necessity	0.171 *	0.135 *	0.213 *
	Overall attitude score	0.212 *	0.188 *	0.235 *
Practices	Adherence to the basic recommendations for COVID-19 prevention	0.143 *	0.121 ^#^	0.168 ^#^
Risk perception	How likely do you think you will contract COVID-19?	0.017 °	0.006 °	0.032 °
	How dangerous do you think COVID-19 is for your health?	0.182 °	0.061 °	0.012 °
	How afraid are you that your family members could contract COVID-19?	0.017 °	−0.002 °	0.052 °
	Overall risk perception score	0.025 °	0.017 °	0.029 °

* *p* < 0.001; # *p* < 0.05; ° *p* > 0.05.

**Table 4:** Multivariate linear regression analysis: four final models (*n* = 751 participants, year 2020).

Model 1 Outcome: Knowledge of Prevention Measures to Protect and Avoid Transmitting COVID-19, from 1 (Very Poor) to 5 (Very Good)				
**Independent Variables (Predictors)**	**Beta**	**t**	***p*** **> |t|**	**95% CI**
One or more risk conditions for severe COVID-19				
No	Ref.	-	-	-
Yes	−0.18	−2.72	0.007	−0.32; −0.05
Smoking habits				
Never smokers	Ref.	-	-	-
Current smokers, less than 10 cigarettes/day	0.07	0.81	0.420	−0.11; 0.26
Current smokers, 10–20 cigarettes/day	−0.05	−0.24	0.807	−0.49; 0.38
Current smokers, more than 20 cigarettes/day	0.23	2.45	0.015	0.05; 0.42
Former smokers	−0.03	−0.46	0.648	−0.19; 0.12
Health literacy				
Inadequate	Ref.	-	-	-
Problematic	0.11	0.95	0.344	−0.12; 0.34
Sufficient	0.32	2.82	0.005	0.10; 0.54
**Model 2** **Outcome: Overall Attitude Score—from 1 (Low Importance) to 5 (Very Important)**				
**Independent Variables (Predictors)**	**Beta**	**t**	***p*** **> |t|**	**95% CI**
Sex				
Males	Ref.	-	-	-
Female	0.11	2.90	0.004	0.03; 0.18
Age (continuous)	0.003	3.04	0.002	0.001; 0.006
Worker subgroups				
Civil Protection	Ref.	-	-	-
Public employees	−0.13	−3.27	0.001	−0.21; −0.05
Health literacy				
Inadequate	Ref.	-	-	-
Problematic	0.17	2.44	0.015	0.03; 0.31
Sufficient	0.33	4.77	<0.001	0.19; 0.46
**Model 3** **Outcome: Adherence to the Basic Recommendations for COVID-19 Prevention, from 1 (Never) to 5 (Every Time)**				
**Independent Variables (Predictors)**	**Beta**	**t**	***p*** **> |t|**	**95% CI**
Sex				
Males	Ref.	-	-	-
Female	0.15	3.14	0.002	0.06; 0.24
Worker subgroups				
Civil Protection	Ref.	-	-	-
Public employees	−0.015	−2.89	0.004	−0.25; −0.05
Smoking habits				
Never smokers	Ref.	-	-	-
Current smokers, less than 10 cigarettes/day	−0.002	−0.04	0.970	−0.15; 0.14
Current smokers, 10–20 cigarettes/day	−0.13	−0.75	0.451	−0.48; 0.21
Current smokers, more than 20 cigarettes/day	0.19	2.59	0.010	0.05; 0.34
Former smokers	0.004	0.07	0.941	−0.11; 0.12
**Model 4** **Outcome: Overall Risk Perception Score—from 1 (Not at All) to 5 (Very Much)**				
**Independent Variables (Predictors)**	**Beta**	**t**	***p*** **> |t|**	**95% CI**
Nationality				
Italian	Ref.	-	-	-
Other	0.53	2.13	0.033	0.04; 1.02
One or more risk conditions for severe COVID-19				
No	Ref.	-	-	-
Yes	0.15	2.12	0.034	0.01; 0.28

## Data Availability

The dataset generated and analyzed during the current study is available from the corresponding author on reasonable request.
